# Rare presentation of peripheral primitive neuroectodermal tumor in the maxilla and mandible: A report of two cases

**DOI:** 10.3892/ol.2014.2219

**Published:** 2014-06-03

**Authors:** CHAO WANG, TIAN-TIAN QIU, XIN-FENG YU, MIN XUAN, QUAN-QUAN GU, WEI QIAN, MIN-MING ZHANG

**Affiliations:** Department of Radiology, The Second Affiliated Hospital, Zhejiang University School of Medicine, Hangzhou, Zhejiang 310009, P.R. China

**Keywords:** maxilla, mandible, primitive neuroectodermal tumor, diagnosis, management

## Abstract

Peripheral primitive neuroectodermal tumor (pPNET) is a rare and highly malignant undifferentiated tumor, which presents in infants and young adults. pPNETs in the head and neck region are uncommon and have a varying incidence of occurrence. Peripheral PNETs of the maxilla and mandible are particularly rare. At present, only 16 cases of pPNET of the maxilla and 13 cases of pPNET of the mandible have been reported. The present study describes a case of pPNET of the maxilla in a 16-year-old male and a case of pPNET of the mandible in another 16-year-old male. The present study reports the radiological findings and the clinical courses of the two patients.

## Introduction

Primitive neuroectodermal tumor (PNET) is a rare and highly malignant neoplasm that consists of small, round cells of neural crest origin (1). PNET is further divided into central PNET (cPNET) and peripheral PNET (pPNET), which arise outside the central and sympathetic nervous system. pPNETs are most common in the thoracopulmonary region, for example the Askin tumor, as well as in the abdomen, pelvic cavity and retroperitoneum (2). pPNET of the maxilla and mandible are very rare. At present, to the best of our knowledge, only 16 cases of pPNET of the maxilla and 13 cases of pPNET of the mandible have been reported (3–9). The present study reports a case of maxillary swelling in a 16-year old male and a case of mandibular swelling in another 16-year old male, who were diagnosed with pPNET. Patients provided written informed consent.

## Case report

### Case 1

**Figure 1:**
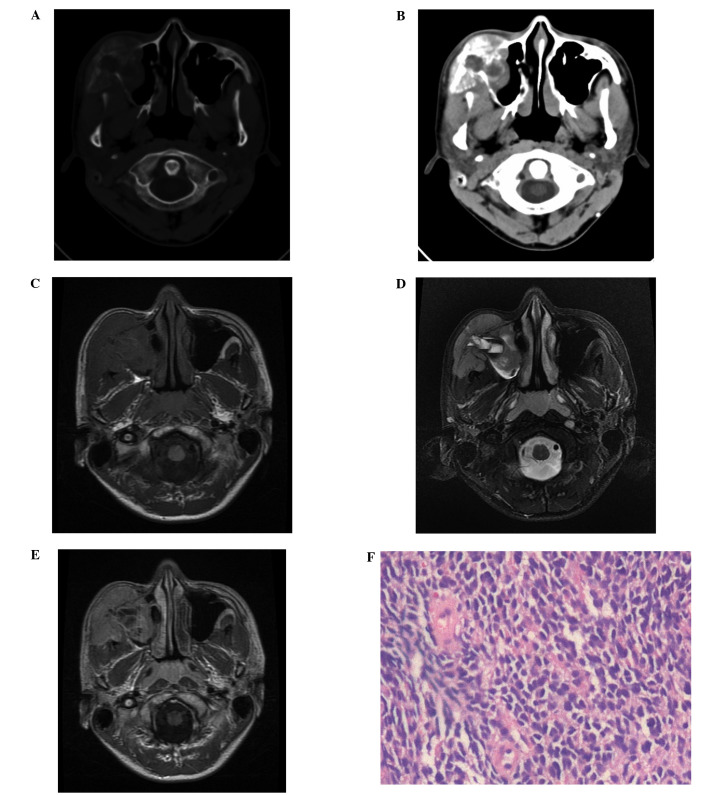
(A) Computed tomography of the head and neck in the bone and (B) soft tissue revealed cortical destruction of the wall of the right maxillary sinus and a sunburst-like periosteal reaction. (C, D and E) Magnetic resonance imaging of the head and neck revealed a soft tissue mass arising from the right maxilla, which occupied the right maxillary sinus. The solid section of the tumor was (C) isointense to the normal muscle on the T1WI and (D) heterogeneous hyperintense on the T2WI. (E) Marked heterogeneous enhancement with a necrotic area was identified following the intravenous administration of gadolinium on the contrast-enhanced T1WI. (F) Poorly differentiated tumor cells with small, blue, round-to-oval nuclei and scant cytoplasm were observed histologically using hematoxylin and eosin staining (magnification, ×10). T1WI, T1-weighted images; T1WI, T2-weighted images.

In June 2011, a 16-year-old male presented to The Second Affiliated Hospital, Zhejiang University School of Medicine (Hangzhou, China) with pain and swelling in the right zygomatic facial region for the previous two months. Upon physical examination, a 4.5×2.5-cm firm, fixed mass with a local sensation of warmth was identified in the right zygomatic facial region. Computed tomography (CT) of the head and neck revealed that the right maxillofacial tumor had caused cortical destruction of the wall of the right maxillary sinus and a sunburst-like periosteal reaction (Fig. 1A and B). Furthermore, the soft tissue mass of the right maxillary sinus was found to be heterogeneous with a low-density necrotic area (Fig. 1B). Magnetic resonance imaging (MRI) of the head and neck revealed a soft tissue mass arising from the right maxilla, which was occupying the right maxillary sinus. The solid section of the tumor was isointense to the normal muscle on the T1-weighted images (T1WI; Fig. 1C) and heterogeneous hyperintense on the T2-WI (T2WI; Fig. 1D). On the contrast-enhanced T1WI, a marked heterogeneous enhancement with a necrotic area was identified following the intravenous administration of gadolinium (Fig. 1E). An ultrasonography-guided percutaneous core needle biopsy using an 18G coaxial cutting needle was performed. The biopsy specimen showed poorly differentiated tumor cells with small, blue, round or oval nuclei and scant cytoplasm (Fig. 1F). Cluster of differentiation (CD)99 (also termed Mic2), vimentin, neuron-specific enolase (NSE), glycoprotein hormones, α-polypeptide (CgA) and S-100 were positive. Creatine kinase (CK), epithelial membrane antigen, myogenin, myogenic differentiation D1, leucocyte common antigen (LCA), desmin and glial fibrillary acidic protein were negative. Based on these histopathological features and the immunohistochemical pattern, the tumor was diagnosed as a pPNET. With a diagnosis of localized pPNET of the right maxilla, the patient commenced treatment with multiagent combination chemotherapy followed by definitive radiation without en bloc resection. This procedure was not performed due to the unfavorable prognosis that is associated with the disease even following en bloc resection. In addition, the extensive cosmetic and functional destruction resulting from resection of the involved section of the maxilla is considered to be unacceptable. The patient demonstrated remission during the 21-month follow-up period.

### Case 2

**Figure 2:**
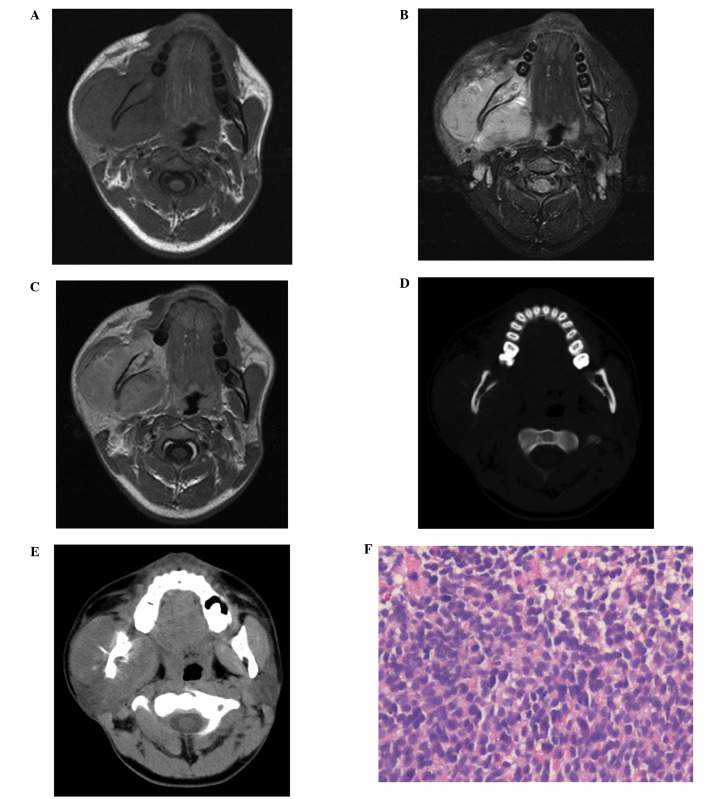
(A, B and C) Magnetic resonance imaging of the head and neck revealed a soft tissue mass arising from the right mandibular ramus, which occupied the right masseter compartment. The tumor was (A) isointense to the normal muscle on the T1WI and (B) hyperintense on the T2WI. (C) The tumor was enhanced heterogeneously following the intravenous administration of gadolinium on the contrast-enhanced T1WI. Computed tomography of the head and neck in the (D) bone and (E) soft-tissue revealed cortical destruction and a sunburst-like periosteal reaction of the right mandibular ramus.(F) A hematoxylin and eosin-stained tumor tissue section revealed small cells, which were diffuse in distribution across the majority of the view (magnification, ×10). T1WI, T1-weighted images; T1WI, T2-weighted images.

In October 2011, a 16-year-old male was transferred to The Second Affiliated Hospital, Zhejiang University School of Medicine with progressive painless swelling of the right mandible, a sensation of numbness and difficulty opening the mouth for one month. The patient was initially admitted to a local hospital with facial cellulitis due to the patient’s prolonged problem with dental caries and swelling of the right side of the chin. The patient was treated with empirical intravenous antibiotic treatment for a presumed infection of dental origin. However, the painless facial swelling became aggravated and the seventh and eighth right mandibular molars were subsequently extracted. The painless facial swelling continued for approximately one week, following which the patient was transferred to The Second Affiliated Hospital, Zhejiang University School of Medicine. Upon examination, the patient showed a firm non-tender fixed mass (size, 6.9×6.9 cm) with a local sensation of warmth on the right lateral aspect of the chin, wrapped around the angle of the mandible. A sensation of numbness was identified in the right lower lip and in the skin of the chin. Following admission to hospital, further imaging analyses of the head and neck were performed. MRI of the head and neck revealed a soft tissue mass arising from the right mandibular ramus, which was occupying the right masseter compartment. The tumor was isointense to the normal muscle on the T1WI (Fig. 2A) and hyperintense on the T2WI (Fig. 2B). On the contrast-enhanced T1WI, the mass enhanced heterogeneously following the intravenous administration of gadolinium (Fig. 2C). Bony cortex erosion and the involvement of the right mandibular canal were also evident, as well as bone marrow replacement, which was observed with isointense signal change on the T1WI and the T2WI. CT of the head and neck revealed cortical destruction and a sunburst-like periosteal reaction of the right mandibular ramus (Fig. 2D and E).

An ultrasonography-guided percutaneous core needle biopsy using an 18G coaxial cutting needle was performed. Histopathologic analysis of the biopsy specimen showed small cells with a diffuse distribution, which occupied the majority of the view (Fig. 2F). CD99, synaptophysin, NSE and CgA were identified to be positive. CK, S-100, LCA and desmin were negative. Based on these histopathological features and the immunohistochemical pattern, the tumor was diagnosed as a pPNET. Oral surgeons were consulted for en bloc resection; however, the tumor was unresectable as it involved the right mandible, parapharyngeal space and right masseter. En bloc resection of the involved portion of the right mandibular ramus would have caused unacceptable extensive cosmetic and functional destruction. Therefore, the patient was transferred to the radiotherapy department for concurrent chemotherapy and radiotherapy. After three cycles of systemic vein chemotherapy, followed by local treatment in the form of radiation therapy, the right mandibular tumor shrunk significantly. The patient received a further three cycles of treatment of systemic vein chemotherapy for nearly two months. Despite the treatment, the patient exhibited skull and meninx metastases and succumbed 18 months after diagnosis.

## Discussion

PNET, a subtype of the family of small round-cell malignancies, was first described in 1918 by Stout (10). The PNETs are primarily found in the central nervous system (CNS); however, have also been reported outside the CNS. These pPNETs are most common in the thoracopulmonary region, abdomen, pelvic cavity and retroperitoneum (2). Due to their rarity, insidious clinical symptoms and variable locations, the accurate diagnosis of pPNETs poses a challenge for clinicians and radiologists.

pPNETs are primarily exhibited in infants and young adults (11). Furthermore, a previous study has reported a marginal male predominance (8). The head and neck region is an unusual location for pPNET (7). The pPNETs are a subtype of the family of small, round-cell malignancies. pPNETs of the maxilla and mandible are particularly rare and following a review of the literature, only 16 cases of pPNET of the maxilla and 13 cases of pPNET of the mandible were identified (3–9).

Pathologically, PNETs represent a transition between neoplastic Schwann cells, neuroblasts and possibly paraganglionic elements (6). It is important that this diagnosis is considered in infants and young adults who present with small round-cell tumors of the bone and soft tissue. The differential diagnosis of small round-cell tumors in the head and neck includes malignant lymphoma, leukemia, neuroblastoma, leiomyosarcoma, rhabdomyosarcoma, undifferentiated carcinoma and pPNET-Ewing sarcoma (4,5). In the two cases described in the present study, the final diagnosis of pPNET was based on immunohistochemistry.

The imaging features of pPNETs are non-specific with regard to the differentiation of pPNETs from other types of bone and soft tissue tumors (3,7). On CT, PNETs usually appear isodense or slightly hypodense when compared with the normal muscle and tumor calcifications are uncommon. Central hypodense areas, consistent with tumor necrosis and cystic change, are found in large tumors. Furthermore, hyperdensities that are consistent with hemorrhage are occasionally observed. Almost all of these tumors demonstrate heterogeneous enhancement with intravenous contrast agents. On MRI scans, the majority of pPNETs are isointense or slightly hyperintense on T1WI and hyperintense on T2WI. Furthermore, the tumor is often heterogeneously marked following the intravenous administration of gadolinium. When they are present, cystic necrotic components and hemorrhagic changes are usually obvious on MRI. Imaging in cases of pPNET of the maxilla and the mandible have been reported to show cortical destruction (4,5) and may exhibit a periosteal reaction (8). In the present cases, the use of MRI and CT predicted whether the tumor was resectable, detected distant metastases and assessed the tumor response to treatment. pPNETs progress rapidly and the prognosis of pPNETs is generally unfavourable. The incidence of distant metastases to the lung, liver, bone, meninx and lymph nodes may be high (4,6). In the present study, in Case 2, the right mandibular tumor shrank significantly following numerous cycles of chemotherapy and radiotherapy; however, the patient demonstrated skull and meninx metastases. Ultrasonography-guided percutaneous core needle biopsy is less invasive compared with open surgical biopsy. Real-time ultrasonographically-guided percutaneous core needle biopsy allows precise needle position and avoids vascular damage, as well as ensuring that the needle biopsy is performed quickly and safely on soft tissue masses around superficial bone lesions (8).

At present, there is no consensus with regard to the guidelines for the treatment of pPNET, due to its rare occurrence, particularly in the head and neck. Due to its similarity to Ewing sarcoma, surgical resection followed by adjuvant radiotherapy at a dose of 45–70 Gy, as well as multiagent chemotherapy if possible, is necessary to improve patient survival (3–7). However, in accordance with the studies by Yeh *et al* (8) and Mohindra *et al* (12), in the present study, the two patients were treated with concurrent radiotherapy and chemotherapy without surgical resection based on the unfavorable prognosis associated with the disease even after en bloc resection, and the unacceptable extensive cosmetic and functional destruction that would be caused following the resection of the involved portion of the maxilla or mandible. Close cooperation between surgeons, oncologists, radiotherapists and radiologists is required for the treatment of pPNET. Furthermore, close follow-up with regular radiographic examinations for at least five years is imperative.

In conclusion, pPNETs of the maxilla or mandible are particularly rare (3–9); thus, the differential diagnosis of pPNET is important. CT and MRI are useful for delineating the extent of the tumor, identifying distant metastases, predicting resectability and monitoring treatment. Therefore, a combination of surgical resection, adjuvant radiotherapy and chemotherapy is the recommended treatment choice.

## References

[1] Schulman H, Newman-Heinman N, Kurtzbart E, Maor E, Zirkin H, Laufer L (2000). Thoracoabdominal peripheral primitive neuroectodermal tumors in childhood: radiological features. Eur Radiol.

[2] Ibarburen C, Haberman JJ, Zerhouni EA (1996). Peripheral primitive neuroectodermal tumors. CT and MRI evaluation. Eur J Radiol.

[3] Jones JE, McGill T (1995). Peripheral primitive neuroectodermal tumors of the head and neck. Arch Otolaryngol Head Neck Surg.

[4] Kao SY, Yang J, Yang AH, Chang KW, Chang RC (2002). Peripheral primitive neuroectodermal tumor of the maxillary gingivae with metastasis to cervical lymph nodes: report of a case. J Oral Maxillofac Surg.

[5] Sun G, Li Z, Li J, Wang C (2007). Peripheral primitive neuroectodermal tumour of the maxilla. Br J Oral Maxillofac Surg.

[6] Hormozi AK, Ghazisaidi MR, Hosseini SN (2010). Unusual presentation of peripheral primitive neuroectodermal tumor of the maxilla. J Craniofac Surg.

[7] Zhang WD, Chen YF, Li CX, Zhang L, Xu ZB, Zhang FJ (2011). Computed tomography and magnetic resonance imaging findings of peripheral primitive neuroectodermal tumors of the head and neck. Eur J Radiol.

[8] Yeh CH, Yeow KM, Chu SY (2011). Imaging findings in mandibular primitive neuroectodermal tumour: a report of a rare case and review of the literature. Dentomaxillofac Radiol.

[9] Bakhshi S, Pathania S, Mohanti BK, Thulkar S, Thakar A (2011). Therapy and outcome of primitive neuroectodermal tumor of the jaw. Pediatr Blood Cancer.

[10] Stout AP (1918). A tumor of the ulnar nerve. Proc NY Pathol Soc.

[11] Dick EA, Mchugh K, Kimber C, Michalski A (2001). Imaging of non-central nervous system primitive neuroectodermal tumours: diagnostic features and correlation with outcome. Clin Radiol.

[12] Mohindra P, Zade B, Basu A (2008). Primary PNET of maxilla: an unusual presentation. J Pediatr Hematol Oncol.

